# Koch’s Lymph in Bovine Tuberculosis

**Published:** 1891-04

**Authors:** 


### KOCH'S LYMPH IN BOVINE TUBERCULOSIS.

It appears odd that with the great prevalence of tuberculosis in cattle, that no experiments should have been made, or at least have been reported in regard to the action of Koch's Lymph, in those animals affected with the disease. The Veterinary Department of the University of Pennsylvania has taken the lead in investigations of this character. A commission has been appointed consisting of a number of members of the Veterinary Faculty, which have been furnished with Koch's Lymph, and has been impowered to obtain tuberculous cattle. We will look with interest for its report to learn if this remedy will produce the same reaction in cattle as in man, and if it will have any value as a means of diagnosis, or as a therapeutic agent.

